# Microbial Inoculants Alleviate Continuous Cropping Obstacles in Eggplant Through Soil Properties and Rhizosphere Microbiota

**DOI:** 10.3390/microorganisms14030672

**Published:** 2026-03-16

**Authors:** Yuyuan Ma, Jian Ding, Zhixing Nie, Hefu Qian, Jirong Zheng, Hong Wang

**Affiliations:** 1 Vegetable Research Institute, Hangzhou Academy of Agricultural Sciences, Hangzhou 310024, China; 21816050@zju.edu.cn (Y.M.); niezhixing@126.com (Z.N.); topzheng2003@163.com (J.Z.); 2 Zhejiang Agricultural Technology Extension Center, Hangzhou 310020, China; dfding@163.com; 3 Jiande Agricultural Technology Extension Center, Hangzhou 311600, China; 15988165128@139.com

**Keywords:** eggplant, microbial inoculants, continuous cropping, soil fertility, rhizosphere microbiome

## Abstract

Eggplant cultivation faces major challenges from continuous cropping obstacles, which degrade soil health and limit sustainable production. Microbial inoculants offer a promising strategy for addressing such issues by modifying the soil environment and rhizosphere ecology. In this study, a field experiment was conducted to evaluate the effects of three bacterial inoculants, including *Bacillus zhangzhouensis* (BF1), *Bacillus mobilis* (BF2), and *Zhihengliuella halotolerans* (BF3), on soil properties, microbial community structure, and crop performance in a continuously cropped eggplant system. The results showed that three inoculants exerted strain-specific effects: BF1 significantly promoted eggplant vegetative growth and yield, increasing plant height by 32.1%, stem diameter by 28.7%, and total yield by 142.4% relative to the control; BF3 selectively improved fruit quality and soil nutrient status, elevating eggplant fruit total amino acid, soluble protein, and soluble sugar contents by 68.9%, 52.3%, and 41.2%, respectively, and increasing soil organic carbon (SOC), total nitrogen (TN), and available nitrogen (AN) by 13.73%, 18.03%, and 84.92% compared with the control. BF2 showed limited efficacy relative to the control. All inoculants enhanced the abundance of beneficial bacteria and reshaped the rhizosphere microbial community structure. The findings demonstrate the potential of strain-specific microbial inoculants to alleviate continuous cropping obstacles and promote sustainable eggplant production.

## 1. Introduction

Eggplant (*Solanum melongena* L.) is a globally cultivated vegetable crop highly valued for its nutritional and economic importance [1,2,3]. With the increasing scarcity of arable land and ongoing agricultural industrialization, continuous eggplant cropping—the practice of cultivating eggplant repeatedly on the same land across consecutive growing seasons—has become a common practice worldwide. However, prolonged monoculture often leads to soil sickness syndrome, a phenomenon attributed to multiple biotic and abiotic factors [4]. These include soil quality degradation, frequent pest and pathogen outbreaks, and structural imbalances in soil microbial communities, marked by reduced abundances of beneficial taxa and the concurrent enrichment of soil-borne pathogenic fungi [5,6]. Such imbalances do not merely represent shifts in community composition; they directly impair key soil ecological processes, including nutrient cycling and pathogen suppression, which in turn lead to measurable reductions in crop nutrient uptake efficiency, weakened disease resistance, and ultimately decreased yield and quality of continuously cropped crops [7,8]. These issues collectively pose a significant challenge to the sustainable production of eggplant.

Long-term monoculture significantly deteriorates soil physicochemical properties, creating an environment conducive to continuous cropping obstacles. The biased uptake of specific nutrients by a single crop results in soil nutrient imbalance, altering the evolutionary trajectory of microbial communities [9]. A key biological driver of this degradation is the gradual accumulation of root exudates, particularly autotoxic phenolic acids such as benzoic acid and vanillic acid [10]. As cropping years increase, the soil accumulation of these autotoxins is exacerbated by acidification [11]. This accumulation exerts dual pressures on the rhizosphere ecosystem: it directly inhibits beneficial microorganisms involved in nutrient cycling and soil structure improvement [12], while simultaneously providing a carbon source that promotes the proliferation of soil-borne pathogens [13,14]. Consequently, these physicochemical and biochemical changes collectively reshape the microbial habitat. They induce shifts in microbial community composition and soil enzyme activities, disrupt nutrient cycling processes, and ultimately facilitate the recruitment of pathogenic microbes into more favorable ecological niches, thereby destabilizing the soil ecosystem and impairing plant growth [7]. Collectively, these stresses pose persistent threats to plant growth and development [15]. Soil microorganisms serve as sensitive bioindicators of soil health and quality, rapidly responding to environmental changes and playing fundamental roles in soil ecosystem functioning [16,17,18]. Accumulating evidence indicates that long-term continuous cropping substantially disrupts soil microbial diversity and community structure [19,20]. With the increasing duration of continuous cropping, the rhizosphere microbial community undergoes substantial alterations in both diversity and richness [21]. Specifically, the abundance of functional taxa such as aerobic and nitrogen-fixing bacteria decreased markedly, whereas the diversity indices of both soil fungi and bacteria declined. This decline in diversity, particularly the loss of key functional groups, undermines critical soil ecosystem services. For instance, the reduction in nitrogen-fixing and nitrifying bacteria directly impairs soil nitrogen supply and cycling efficiency, while a diminished and less diverse microbial consortium weakens the soil’s innate suppressiveness against pathogens and its resilience to environmental stress [22,23]. Disruption of the original soil microbial community structure ultimately impairs plant growth.

Soil microbial taxa at the phylum level are the core components driving soil ecological function, and their structural composition and abundance dynamics are closely linked to soil fertility maintenance, nutrient cycling and plant health regulation in continuous cropping systems. Pseudomonadota is a key phylum involved in soil nitrogen and phosphorus cycling, and its functional strains can enhance plant nutrient uptake and induce plant systemic disease resistance [24,25]; Bacillota exhibits strong metabolic activity, as its representative taxa are capable of restoring soil organic carbon mineralization, enhancing microbial respiration, and ultimately contributing to the recovery of soil function in intensive agricultural systems [26]. Planctomycetota is closely related to soil organic carbon sequestration and micro-aggregate formation, and can improve soil structural stability [27]; Acidobacteriota is a typical oligotrophic phylum whose abundance is an important indicator of soil ecosystem stability and organic matter accumulation level [28]. Gemmatimonadota participates in soil carbon and nitrogen metabolism and has a regulatory effect on the balance of rhizosphere microbial communities [29]; Chloroflexota is a common phylum in continuous cropping soil, and its excessive enrichment is often associated with the deterioration of the soil micro-ecological environment and the occurrence of continuous cropping obstacles [30]. The dynamic changes in these dominant phyla are the direct reflection of the response of the rhizosphere microbial community to continuous cropping stress and microbial inoculant regulation, and their structural optimization is the key to alleviating eggplant continuous cropping obstacles.

Microbial diversity and an appropriate community composition are essential for maintaining soil health and promoting plant growth. This aligns with the “insurance hypothesis” of microbial ecology, which posits that higher microbial diversity enhances ecosystem stability and functionality by providing functional redundancy, thereby ensuring the maintenance of key ecological processes even when individual taxa are impaired by environmental stress [31,32]. Furthermore, intricate, bidirectional mutualistic interactions exist between plant root systems and soil microorganisms, forming a dynamic rhizosphere microecosystem that is critical for plant adaptation and soil health preservation [33]. Soil bacteria and fungi influence plant health through both direct and indirect pathways. Beneficial bacteria, such as *Rhizobia* and *Bacillus* species, enhance nutrient acquisition and synthesize bioactive compounds that promote plant growth [34,35]. In turn, plant roots profoundly shape the rhizosphere microbial community by releasing exudates and secondary metabolites, which act as chemoattractants or repellents to select specific microbial taxa, thereby regulating microbial diversity and community composition [36]. These intimate plant–microbe interactions facilitate plant adaptation to natural environments, including continuous cropping systems characterized by abiotic stresses such as nutrient imbalance and soil acidification, along with biotic stresses including pathogen invasion [37]. Furthermore, intricate interactions exist between plant root systems and soil microorganisms. Soil bacteria and fungi both directly and indirectly influence plant health, and plant roots profoundly shape the rhizosphere microbial community by releasing exudates and secondary metabolites [38]. These interactions facilitate plant adaptations to natural environments, including continuous cropping systems [39]. Elucidating the diversity and composition of microbial communities in soils under continuous cropping conditions may enhance our understanding of plant performance under such agricultural regimes.

Crop rotation, adopted as an alternative to continuous cropping, effectively mitigates the soil sickness induced by the latter, although its implementation may create economic challenges and increase management complexity for farmers. In agricultural practice, chemical fertilizers are commonly used to mitigate soil sickness caused by continuous cropping, but they often induce soil acidification, salinization, and nutrient imbalance, thereby threatening soil productivity and the stability of resident microbial communities [40]. In contrast, accumulating evidence indicates that organic amendments offer a more sustainable alternative, as they effectively enhance soil fertility, suppress soil-borne pathogens, and improve the structure of soil microbial communities, thus alleviating the adverse effects of monoculture [41,42]. Microbial-based biofertilizers, which are recognized as sustainable alternatives for enhancing soil fertility, have garnered significant attention because of their potential to preserve soil health and improve crop product quality. Microbial inoculants are formulations containing highly active functional microorganisms designed to enhance plant nutrient uptake, improve crop growth vigor, and ameliorate the soil physical structure. Specifically, these products contribute to nutrient activation in the soil, regulation of pH balance, improvement of soil aeration, and mobilization of fixed nutrients, thereby significantly increasing nutrient use efficiency and reducing dependence on chemical fertilizers [43,44]. Microbial inoculants are widely employed to optimize the soil microecological environment and effectively enhance systemic disease resistance in crops [45]. Among the diverse functional microbes used in agricultural inoculants, *Bacillus* and *Zhihengliuella* stand out for their remarkable adaptability to stressed agricultural soils and multi-faceted beneficial effects on plants and soil ecosystems, making them ideal candidates for mitigating continuous cropping obstacles in vegetable production. *Bacillus* is a Gram-positive, spore-forming bacterium ubiquitously distributed in soil and rhizosphere ecosystems, and its unique spore structure endows it with strong resistance to adverse soil conditions such as acidification, salinization and nutrient deficiency, allowing it to rapidly germinate, colonize and exert its biological functions in the rhizosphere of crops under stress. *Bacillus zhangzhouensis*, in particular, exhibits a prominent ability to enhance crop salt tolerance through multiple synergistic mechanisms: it synthesizes plant hormones to regulate plant growth and development, maintains cellular osmotic balance to alleviate salt ion damage, promotes the absorption and utilization of soil nutrients by crops, improves photosynthetic efficiency to ensure carbon assimilation under stress, and modulates the plant antioxidant enzyme system to scavenge reactive oxygen species and mitigate oxidative stress induced by salt stress [46,47]. In contrast, *Zhihengliuella halotolerans*, the core strain of BF3 in this study, is a halotolerant bacterium belonging to the phylum Actinomycetota, which is isolated from the rhizosphere of halophytes and has evolved a unique osmotic adjustment system to adapt to high-salt soil environments [48].

In continuous eggplant cropping systems, the strain-specific regulatory effects of single-functional microbial inoculants on rhizosphere microbial community assembly, soil nutrient cycling, and the coupled soil–plant feedbacks remain poorly characterized, with the linkages between inoculant functional traits and eggplant growth/fruit quality outcomes yet to be systematically elucidated. In the present study, we conducted a field experiment to evaluate the effects of three distinct microbial inoculants on eggplant quality, yield, and agronomic traits, as well as to characterize the response of rhizosphere bacterial communities to these inoculants. The three strains were selected for their distinct, targeted traits against eggplant continuous cropping obstacles: (1) *Bacillus zhangzhouensis* (BF1) produces antifungal and plant growth-modulatory compounds to suppress soil-borne pathogens and boost plant vigor [49]; (2) *Bacillus mobilis* (BF2) was screened for its superior plant growth-promoting traits in our preliminary assays; (3) *Zhihengliuella halotolerans* (BF3) tolerates 6% NaCl and decomposes organic matter, thus adapting to salinized soils and effectively activating soil nitrogen and carbon cycling [50]. Collectively, this study may contribute to the development of targeted strategies for soil microbiome management and the mitigation of continuous cropping obstacles by analyzing the shifts in bacterial community composition induced by different microbial treatments.

## 2. Materials and Methods

### 2.1. Site Description

The eggplant cultivation experiment was conducted at the Zhijiang Experimental Base of the Hangzhou Academy of Agricultural Sciences (30°15′ N, 120°09′ E), located in Hangzhou, China. The soil was loamy. Prior to the experiment, soil samples (0–20 cm depth) were collected from 15 randomly selected points across the field and mixed thoroughly for the determination of baseline physicochemical properties. The pre-experiment soil properties were as follows: pH 8.064 and electrical conductivity (EC) 884.96 ± 140.19 μs/cm.

### 2.2. Strain Isolation and Inoculant Preparation

The three functional strains (*Bacillus zhangzhouensis*, *Bacillus mobilis*, and *Zhihengliuella halotolerans*) were isolated from the rhizosphere soil of halophytes (*Suaeda salsa* and *Tamarix ramosissima*) sampled on 23 June 2023, in Haixi Mongolian and Tibetan Autonomous Prefecture, Qinghai Province (36°37′ N, 95°11′ E).

Strain culture and preparation were performed as follows: Glycerol-preserved strains were streaked onto trypticase soy broth (TSB) solid plates for activation. Single colonies were picked and inoculated into liquid TSB medium to prepare seed cultures. The seed cultures were then inoculated into fresh liquid TSB medium at a 1% (*v*/*v*) inoculation ratio for expansion culture, and the bacterial suspension was collected when the OD_600_ value reached 1.0. The TSB medium used in this experiment was purchased from Haibo Biotechnology Co., Ltd., Qingdao, China.

### 2.3. Field Experiment

The experiment adopted a randomized complete block design (RCBD) with three replicates per treatment. Each plot had an area of 4.8 m^2^ (3 m × 1.6 m), with a row spacing of 0.6 m and plant spacing of 0.4 m, accommodating 20 eggplant plants per plot. Plot spacing was set at 1 m to avoid cross-contamination between treatments. Four treatments were established in the experiment: (1) control (CK), with no fertilizer added; (2) BF1, a microbial inoculant formulation primarily based on *Bacillus zhangzhouensis* as the functional strain; (3) BF2, a microbial inoculant formulation primarily based on *Bacillus mobilis* as the functional strain; and (4) BF3, a microbial inoculant formulation primarily based on *Zhihengliuella halotolerans* as the functional strain. A 50 mL aliquot of each bacterial inoculant (2 × 10^8^ CFU mL^−1^ in sterile TSB) was diluted 20-fold with sterile deionized water and applied to the seedling tray at a rate of 1 L per tray through root irrigation, resulting in a final inoculation concentration of 1.2 × 10^6^ CFU g^−1^ dry rhizosphere soil. All bacterial inoculants were prepared as liquid formulations with the original concentration of 2 × 10^8^ CFU mL^−1^, and the CK group received an equal volume of sterile TSB medium diluted 20-fold with sterile water.

This experiment was conducted in a continuous eggplant-cropping system and lasted four months, from seedling transplantation on 15 April 2025, to the final harvest in August 2025. Inoculants were applied to the rhizosphere soil during transplantation, while the control plots received an equal volume of sterile liquid medium (consistent with the inoculant carrier) to eliminate confounding effects of liquid application.

### 2.4. Determination of Agronomic Traits, Fruit Quality, and Yield

At maturity, five plants were randomly sampled from each plot to measure agronomic traits, including plant height and stem diameter, and the mean values were calculated for each parameter. From each treatment, five ripe fruits were collected for biochemical analysis to determine the total amino acid, soluble protein, and soluble sugar content. Yield was assessed by harvesting all eggplants from a defined area of consistent size within each plot, with a total of four harvests conducted at five-day intervals.

Total amino acid content was determined by liquid chromatography-mass spectrometry (LC-MS) [51]. Harvested eggplant fruits were snap-frozen in liquid nitrogen and lyophilized for 72 h (LyoQuest-85, Telstar Technologies, Barcelona, Spain). The dried samples were pulverized into a homogeneous powder (TissueLyser II, QIAGEN, Hilden, Germany). For metabolite extraction, 0.1 g of the powder was mixed with 1 mL of 0.5 M hydrochloric acid, subjected to ultrasonic-assisted extraction, and centrifuged at 20,000× *g* for 20 min (3–18 KS, Sigma, Osterode am Harz, Germany). The supernatant was filtered through a 0.22 µm aqueous filter membrane, and 5 µL of the filtrate was injected into an LC-MS system (Agilent 1290-6460, Agilent Technologies Inc., Santa Clara, CA, USA) for quantitative analysis. Chromatographic separation was performed on an Agilent InfinityLab Poroshell 120 HILIC-Z column (2.1 × 100 mm, 2.7 µm) at 25 °C, with mobile phases A (water:200 mM ammonium formate = 9:1, *v*/*v*) and B (acetonitrile:200 mM ammonium formate = 9:1, *v*/*v*) (final ammonium formate concentration: 20 mM for both phases) at a flow rate of 0.5 mL/min. Mass spectrometry conditions: multi-response monitoring (MRM) mode, electrospray ionization (ESI) positive ion mode, drying gas temperature 330 °C (13.0 L·min^−1^), atomizer pressure 35 psi, sheath gas temperature 390 °C (12 L·min^−1^), and capillary voltage 1500 V.

The soluble protein content was determined using the Coomassie Brilliant Blue method [52]. Fruit tissue (0.2 g) was cut into pieces and put into a mortar, ground thoroughly with a small amount of chilled phosphate-buffered saline (PBS, 0.01 M, pH 7.4), and centrifuged quickly at 10,000× *g* for 10 min at 4 °C. One milliliter of the supernatant was absorbed, and 5 mL of Coomassie Brilliant Blue G-250 solution was added, mixed uniformly, and allowed to stand at room temperature for 5 min before measuring the absorbance with a colorimeter at 595 nm.

The soluble sugar content was determined using the anthrone-sulfuric acid method [53]. Briefly, 0.1 g of fresh eggplant fruit tissue was accurately weighed and ground into a homogenate with 5 mL of distilled water. The homogenate was subjected to a boiling water bath for 30 min to fully extract soluble sugars, then filtered to collect the filtrate. The filtrate was transferred to a 25 mL volumetric flask, and the volume was adjusted to the marked line with distilled water to obtain the standardized extract. For color development, 500 μL of the standardized extract was pipetted into a clean test tube, followed by the addition of 1.5 mL of distilled water, 0.5 mL of anthrone-ethyl acetate reagent (prepared by dissolving 1 g of anthrone in 50 mL of ethyl acetate and mixing thoroughly), and 5 mL of concentrated sulfuric acid. After vortexing vigorously to mix evenly, the test tube was immediately placed in a boiling water bath for 1 min, then removed and allowed to cool naturally to room temperature. The absorbance of the reaction mixture was measured at 630 nm using a spectrophotometer. A standard curve was constructed using pure sucrose as the standard substance following the same experimental procedure, and the concentration of soluble sugars in the eggplant fruit samples (C, μg/mL) was calculated based on the standard curve. The final soluble sugar content was expressed as mg g^−1^ fresh weight (FW).

### 2.5. Analysis of Soil Microbial Diversity

The rhizosphere and non-rhizosphere soils were collected from five randomly selected plants per treatment at the fruit harvest stage, with each treatment comprising five biological replicates. Rhizosphere soil was collected using the shake-off method with minor modification [54]: roots with adhering soil (≤2 mm from the root surface) were gently placed in sterile centrifuge tubes, shaken vigorously for 1 min, and the soil suspension was collected by centrifugation (5000× *g*, 10 min) to obtain rhizosphere soil samples. Non-rhizosphere soil samples were collected from the 0–20 cm soil layer in the inter-row spaces, away from plant roots, to avoid rhizosphere effects.

The experimental layout for soil sampling included two soil niche types (rhizosphere/non-rhizosphere) × four inoculant treatments (CK, BF1, BF2, BF3), resulting in eight total sample groups, abbreviated as follows: R-CK, R-BF1, R-BF2, R-BF3 (rhizosphere soil under each treatment) and NR-CK, NR-BF1, NR-BF2, and NR-BF3 (non-rhizosphere soil under each treatment).

Microbial DNA was extracted from 0.5 g of homogenized soil using the NucleoSpin^®^ Soil Kit (Macherey-Nagel, Düren, Germany) following the manufacturer’s standard protocol with minor modifications: briefly, soil samples were mixed with the kit-provided CTAB-based lysis buffer supplemented with 2% polyvinylpyrrolidone (PVP-40) to enhance microbial cell lysis and eliminate humic acid contaminants, incubated at 65 °C for 1 h with gentle inversion every 15 min, and then subjected to bead-beating using a TissueLyser II (QIAGEN, Hilden, Germany) at 25 Hz for 2 min to further disrupt recalcitrant microbial cells. Subsequent protein precipitation, DNA binding to spin columns, washing, and elution steps were performed strictly according to the kit instructions. DNA quality was evaluated using a three-dimensional assessment system to ensure suitability for downstream sequencing, including 1% agarose gel electrophoresis (120 V, 30 min) to verify fragment integrity (target band size: ~20 kb, free of smearing), a NanoDrop 2000 spectrophotometer (Thermo Fisher Scientific, Waltham, MA, USA) to assess purity (A260/A280 ratio: 1.8–2.0; A260/A230 ratio: >1.5, indicating low levels of humic acid and protein contamination), and a Qubit 4.0 Fluorometer (Thermo Fisher Scientific) to accurately quantify DNA concentration. Only high-quality microbial DNA samples meeting all three criteria were diluted with sterile nuclease-free water to 1 ng/μL for downstream PCR amplification and sequencing.

The full-length 16S rRNA gene was amplified from the diluted genomic DNA using the universal primers 27F (5′-AGRGTTYGATYMTGGCTCAG-3′) and 1492R (5′-RGYTACCTTGTTACGACTT-3′). Barcoded PCR products from all samples were pooled at equimolar ratios for library construction using Kinnex long-read sequencing technology (Los Angeles, CA, USA) [55]. The resulting library was purified and subjected to rigorous quality control: concentration was measured with a Qubit^®^ 4.0 Fluorometer (Thermo Fisher Scientific, Waltham, MA, USA), and size distribution was verified using an Agilent 2100 Bioanalyzer (Agilent, Santa Clara, CA, USA), confirming a dominant fragment peak within the expected 1.5–3 kb range. Libraries passing the quality assessment were subsequently sequenced on the Pacific Biosciences (PacBio, Menlo Park, CA, USA) Revio platform via Single Molecule, Real-Time (SMRT) technology to generate high-fidelity long-read sequences. Raw sequencing reads were quality filtered using SeqKit (v2.9.0) to generate high-quality clean tags. These effective tags were subsequently denoised using DADA2 (default) or the Deblur module in QIIME2 to derive amplicon sequence variants (ASVs). For each representative sequence, taxonomic annotation was performed against the SILVA database (http://www.arb-silva.de/, accessed 24 August 2025) using the Mothur algorithm [56].

The bacterial community diversity and composition in each sample were assessed using the Simpson index, Pielou index, Dominance index, Good’s coverage, and Observed features, all of which were calculated using QIIME (version 1.9.1) and visualized in R (version 4.0.3). LEfSe (linear discriminant analysis effect size) was performed using the lefser package in R (version 4.0.3) to identify statistically significant biomarkers and elucidate metagenomic features. Rarefaction curves, principal component analysis (PCA), redundancy analysis (RDA), and community heatmaps were generated with the vegan package (version 2.6-4) in the same R environment.

### 2.6. Analysis of Physicochemical Soil Properties

Soil samples were collected at the fruiting stage for the determination of soil nutrient contents as follows: Soil total phosphorus (TP) content was determined using the molybdenum blue colorimetric method [57]; TN and AN content were determined using the Kjeldahl method, followed by colorimetric analysis [58]; soil organic matter (SOM) content was determined by potassium dichromate oxidation and back titration of excess potassium dichromate using an ammonium ferrous sulfate solution [59]; SOC was determined using the Walkley–Black dichromate oxidation method [60]; and the soil total potassium (TK) content was determined using flame photometry [61].

### 2.7. Statistical Analysis

All statistical analyses were performed using IBM SPSS Statistics (version 20.0; IBM Corp., Chicago, IL, USA) and R software (version 4.0.3). IBM SPSS Statistics was selected for one-way analysis of variance (ANOVA) and post hoc significance tests due to historical continuity and standardized data processing within our team’s agricultural research framework, which ensures consistency in methodological reporting across our related publications. Statistical significance was defined as *p* < 0.05. Data visualization, including histograms and heat maps, was performed using GraphPad Prism software (version 8.0; GraphPad Software, San Diego, CA, USA).

## 3. Results

### 3.1. Microbial Inoculants Enhance Eggplant Growth, Yield, and Quality

**Figure 1:**
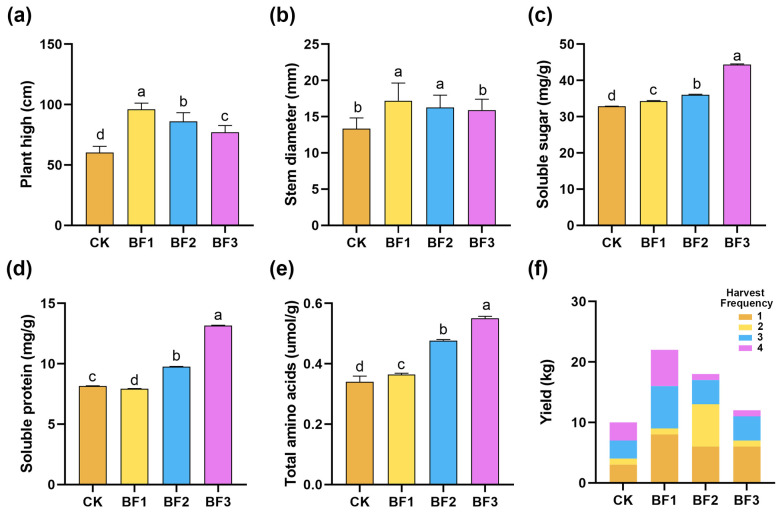
Yield and agronomic characteristics of eggplant under different inoculant treatments. (**a**) Plant height; (**b**) stem diameter; (**c**) soluble sugar content; (**d**) soluble protein content; (**e**) total amino acid content; (**f**) yield (stratified by harvest times). Five replicates for each treatment. All data are presented as mean ± SEM (*n* = 5 biological replicates). Different letters above bars indicate significant differences among treatments (*p* < 0.05). CK, control (no fertilizer added); BF1, inoculant based on *Bacillus zhangzhouensis*; BF2, inoculant based on *Bacillus mobilis*; BF3, inoculant based on *Zhihengliuella halotolerans*.

The application of different microbial inoculants significantly influenced the agronomic traits, yield, and physiological quality of eggplants (Figure 1). Among all treatments, BF1 resulted in the highest plant height (96.06 ± 3.25 cm) and the thickest stem diameter (17.19 ± 0.82 mm), whereas the control group (CK) exhibited the lowest values for most of the measured parameters. The yield showed a clear response to the application of microbial inoculants, with BF1 producing the highest total yield (23.15 ± 1.87 kg), whereas the CK total yield was only 9.55 ± 0.91 kg. In terms of fruit quality traits, BF3 led to the most notable improvements, achieving the highest total amino acid content (0.551 µmol/g), soluble protein (13.1738 mg/g), and soluble sugar (44.361 mg/g). BF1 and BF2 significantly promoted vegetative growth and increased yield, while BF3 significantly improved the main fruit quality indices.

### 3.2. Microbial Inoculants Improve Rhizosphere Microbial Diversity in Eggplant

Rhizosphere and non-rhizosphere soil samples were analyzed separately to compare the differential effects of microbial inoculants on the microbial communities in the two distinct soil niches.

**Figure 2:**
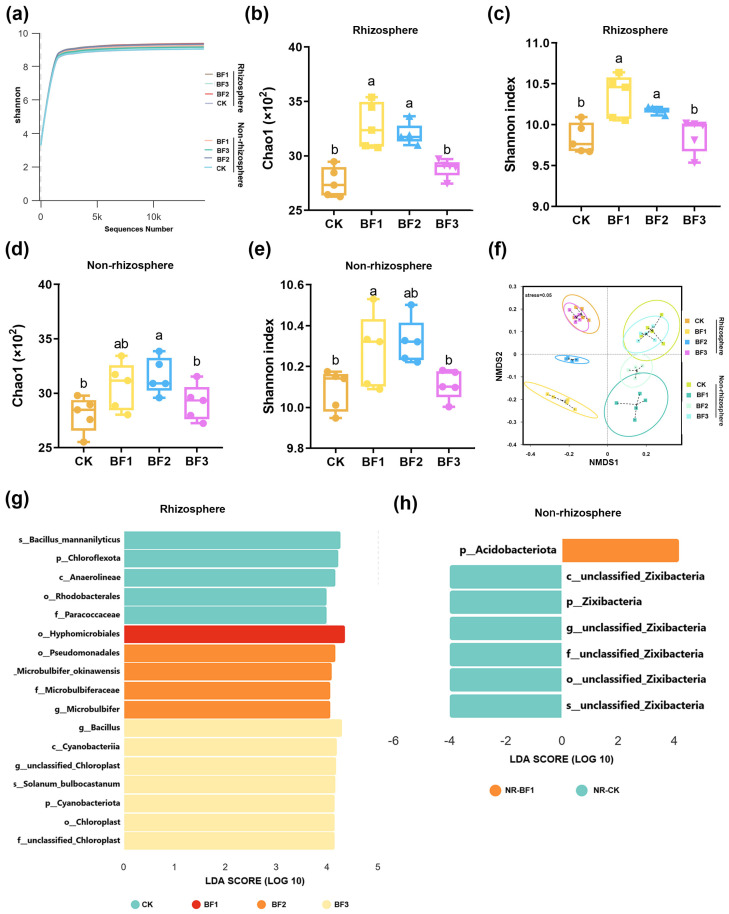
Effects of microbial inoculants on rhizosphere and non-rhizosphere soil microbiomes. (**a**) Rarefaction curve for all samples; (**b**) Chao1 richness index of the rhizosphere microbial community; (**c**) Shannon evenness index of the rhizosphere microbial community; (**d**) Chao1 richness index of the non-rhizosphere soil microbial community; (**e**) Shannon evenness index of the non-rhizosphere soil microbial community; (**f**) β-diversity visualized by non-metric multidimensional scaling (NMDS); (**g**) linear discriminant analysis effect size (LEfSe) analysis of differential microbial taxa in rhizosphere soil (LDA score > 4); (**h**) LEfSe analysis of differential microbial taxa in bulk soil (LDA score > 4). Five biological replicates per treatment; data are shown as mean ± SEM. Different lowercase letters above bars indicate significant differences (*p* < 0.05). CK, control (no fertilizer added); BF1, inoculant based on *Bacillus zhangzhouensis*; BF2, inoculant based on *Bacillus mobilis*; BF3, inoculant based on *Zhihengliuella halotolerans*.

A total of 1,110,215 high-quality bacterial sequence reads were derived from the optimized sequencing data of 40 soil samples, and these sequences were used to evaluate microbial diversity across all treatments. Sequencing coverage exceeded 99.0% for all samples, and the rarefaction curves plateaued beyond 10,000 reads, confirming sufficient depth to capture the majority of species and reliably represent bacterial community structures without substantial discovery of new species upon further sequencing (Figure 2a). Quality-filtered reads were clustered into 4989–6091 bacterial amplicon sequence variants (ASVs) across all samples.

Significant differences in alpha diversity indices (Simpson, Pielou_e, Dominance, Good’s coverage, and Observed features) were detected among the treatments. In rhizosphere soil, microbial inoculant treatments significantly altered both community richness and evenness: BF1 demonstrated the highest bacterial richness (Chao1 index = 3278.8) and diversity (Shannon index = 10.35), followed by BF2 and BF3, whereas CK consistently displayed the lowest values for all indices (Figure 2b,c). Similar trends were reflected in the Simpson and Pielou indices, indicating not only greater species richness but also more uniform community structures in the biofertilizer treatments than in the control (Table 1). In non-rhizosphere soil, the effects of inoculant treatments on microbial α-diversity were relatively limited. Only the Chao1 index in the BF2 treatment and the Shannon index in the BF1 treatment were slightly higher than those in the control (Figure 2d,e).

Non-metric Multidimensional Scaling (NMDS) revealed distinct clusters of soil microbial communities at different sampling sites (Figure 2f). Microbial communities in rhizosphere and non-rhizosphere soil exhibited clear separation along the NMDS1 axis. Within the rhizosphere, samples from the BF1 and BF3 treatments formed distinct clusters separate from the CK, whereas in non-rhizosphere soil, all inoculant-treated samples largely overlapped with CK, with only BF1 showing slight divergence.

Linear discriminant analysis effect Size analysis indicated significant enrichment of specific taxa in rhizosphere soil (Figure 2g). The BF1 treatment showed marked enrichment of taxa, including the order Pseudomonadales and the genus *Microbulbifer* (LDA score > 4), while the CK group was characterized by enrichment of species such as *Bacillus mannanylyticus* and the phylum Chloroflexota. In non-rhizosphere soil (Figure 2h), only minor differential enrichment (the phylum Acidobacteriota) was detected between BF1 and CK, with LDA scores substantially lower than those observed in the rhizosphere. Differential taxon enrichment induced by inoculant treatment was far more pronounced in the rhizosphere than in the non-rhizosphere soil, with notably higher LDA scores in the rhizosphere.

**Figure 3:**
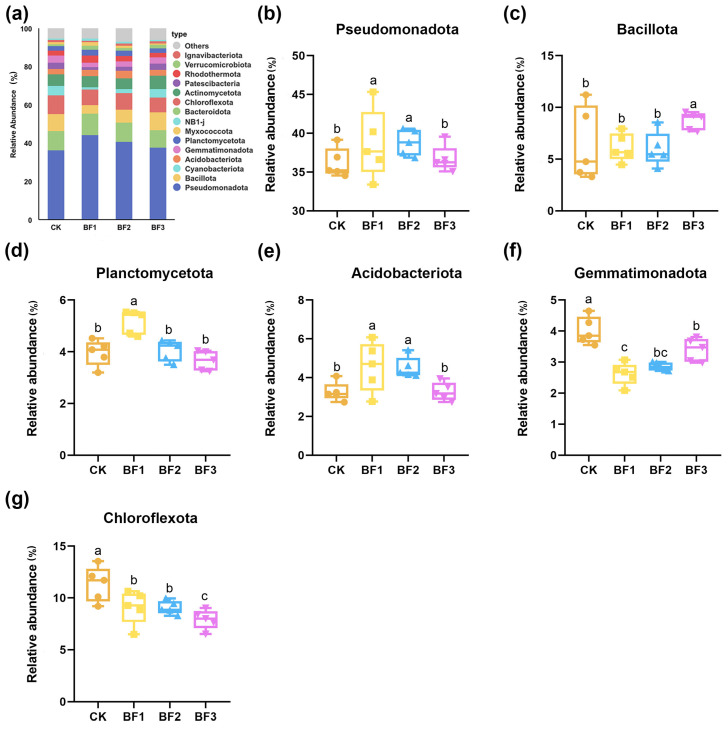
Composition and differences in rhizosphere bacterial communities at the phylum level. (**a**) Relative abundance of dominant bacterial phyla in rhizosphere soil. (**b**) Pseudomonadota; (**c**) Bacillota; (**d**) Planctomycetota; (**e**) Acidobacteriota; (**f**) Gemmatimonadota; (**g**) Chloroflexota. Five replicates for each treatment; all data are mean ± SEM (*n* = 5 biological replicates). Different letters above bars indicate significant differences among treatments (*p* < 0.05). CK, control (no fertilizer added); BF1, inoculant based on *Bacillus zhangzhouensis*; BF2, inoculant based on *Bacillus mobilis*; BF3, inoculant based on *Zhihengliuella halotolerans*.

We systematically analyzed the differences in rhizosphere bacterial communities among the treatment groups (CK, BF1, BF2 and BF3). Initially, the overall community structure was examined at the phylum level. The same ten bacterial phyla constituted the most abundant taxa across all four sampling sites (Figure 3a). The most abundant bacterial phyla were Pseudomonadota, Bacteroidota, Bacillota, Chloroflexota, and Cyanobacteriota. Together with Actinomycetota, these six phyla collectively represented 74.1% to 76.2% of the total bacterial community. The relative abundance of Pseudomonadota was significantly higher in the BF1 and BF2 treatments than that in the CK group (6.77% and 7.24%, respectively; Figure 3b). In contrast, the BF3 treatment led to a marked increase in the relative abundance of Bacillota (35.57%; Figure 3c). The BF1 treatment also resulted in a significantly higher relative abundance of Planctomycetota (25.89%; Figure 3d). With regard to Acidobacteriota, both the BF1 and BF2 treatments showed significantly elevated levels relative to the CK (39.45% and 37.92%, respectively; Figure 3e). Conversely, the relative abundance of Gemmatimonadota was significantly reduced by 34.66% in BF1 compared with CK (Figure 3f). The relative abundance of Chloroflexota was the highest in the CK group and was significantly reduced across all inoculant treatments, with the most pronounced suppression observed under BF3 (Figure 3g). However, the proportions of these bacterial phyla differed among the four treatments. Overall, the use of microbial inoculants resulted in an increase in the abundance of bacterial phyla.

### 3.3. Microbial Inoculant Application Enhances Soil Fertility

**Figure 4:**
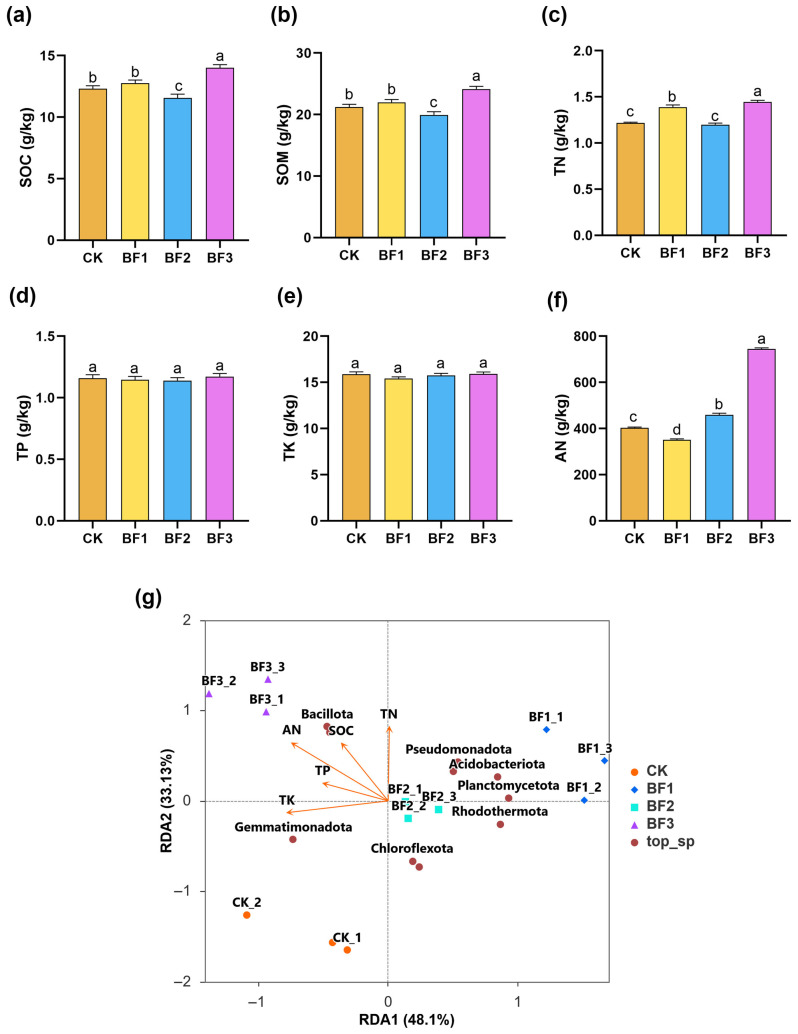
Effects of different inoculant treatments on soil chemical properties. (**a**) Soil organic carbon (SOC); (**b**) soil organic matter (SOM); (**c**) total nitrogen (TN); (**d**) available nitrogen (AN); (**e**) total phosphorus (TP); (**f**) total potassium (TK); (**g**) relationships between soil properties and bacterial community composition based on redundancy analysis (RDA); orange arrows indicate the lengths and angles between explanatory and response variables, reflecting their correlations. bars represent mean values of three replicates ± standard error (SE). Different lowercase letters above bars indicate significant differences among treatments (*p* < 0.05). CK, control (no fertilizer added); BF1, inoculant based on *Bacillus zhangzhouensis*; BF2, inoculant based on *Bacillus mobilis*; BF3, inoculant based on *Zhihengliuella halotolerans*.

Substantial changes in soil physicochemical properties were observed across the treatments (Figure 4). Among the three inoculant treatments, only BF3 exerted a significant positive effect on soil nutrient accumulation compared with CK. Specifically, BF3 significantly increased SOM by 13.76%, SOC by 13.73%, TN by 18.03%, and AN by 84.92%. In contrast, BF1 and BF2 treatments showed no significant differences from CK in most soil nutrient indices (SOC, SOM, TN, TP, TK), with only marginal increases in AN content that did not reach statistical significance. These results indicate that the regulatory effects of microbial inoculants on soil fertility are strain-specific, with BF3 demonstrating the most pronounced capacity to enhance soil carbon and nitrogen pools.

Based on the redundancy analysis (RDA) results, different bacterial inoculant treatments exhibited clear niche differentiation in the ordination space defined by soil environmental factors and microbial community relationships (Figure 4g). The BF3 treatment group clustered independently in the upper-left quadrant of the ordination plot, showing strong positive correlations with SOC, TN, AN, and TP, indicating its pronounced role in promoting soil nutrient accumulation. Bacillota, which were specifically enriched in this region, were identified as the key microbial taxa driving soil nutrient transformation in the BF3 treatment. The BF1 treatment group was distributed in the upper-right area and closely associated with the enrichment of Pseudomonadota and Acidobacteriota. In contrast, the BF2 treatment group clustered near the coordinate origin, with weak correlations with environmental factors, suggesting a relatively limited regulatory effect on soil physicochemical properties. Distinct spatial distribution patterns of inoculant treatments were observed in the RDA ordination plot, with each treatment showing specific correlations with soil environmental factors and dominant microbial taxa.

### 3.4. Microbial Community Structure and Differential Taxa Under Different Inoculant Treatments

**Figure 5:**
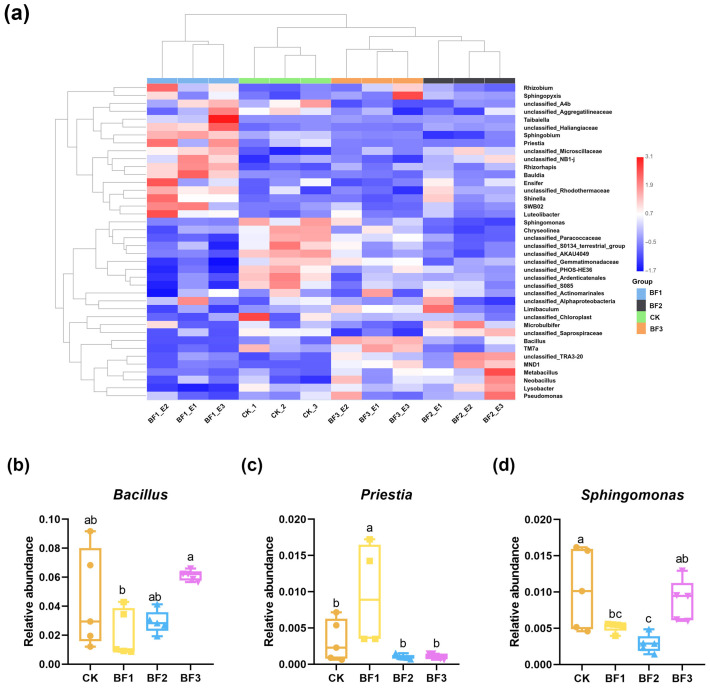

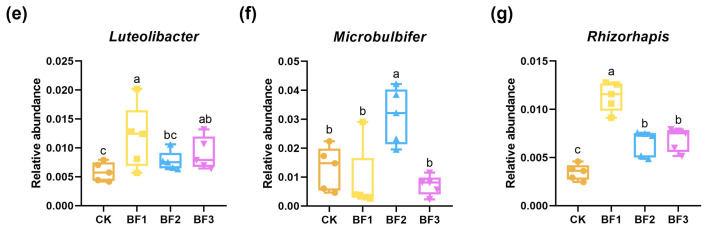
Effects of different inoculant treatments on rhizosphere soil bacterial community composition and core genus relative abundance. (**a**) Comparative analysis of the relative abundance of the top 40 most abundant bacterial genera across the four treatment groups; (**b**) *Bacillus*; (**c**) *Priestia*; (**d**) *Sphingomonas*; (**e**) *Luteolibacter*; (**f**) *Microbulbifer*; (**g**) *Rhizorhapis.* Five replicates for each treatment; all data are presented as mean ± SEM (*n* = 5 biological replicates). Different letters above bars indicate significant differences among treatments (*p* < 0.05). CK, control (no fertilizer added); BF1, inoculant based on *Bacillus zhangzhouensis*; BF2, inoculant based on *Bacillus mobilis*; BF3, inoculant based on *Zhihengliuella halotolerans*. Theorem-type environments (including propositions, lemmas, corollaries, etc.) can be formatted as follows.

A comparative analysis of bacterial composition was conducted among rhizosphere soil samples. This study focused on the top 40 genera identified in each replicate across the four treatment groups, followed by a cluster analysis of these taxa. The resulting heatmap clearly illustrates the relative abundance patterns of these genera. Based on the similarity in bacterial community composition, the four soil sample types were successfully classified into distinct taxonomic groups. Analysis of the heatmap revealed a clear segregation of bacterial genera into two distinct clusters: one comprising samples CK, BF2, and BF3 and the other consisting solely of BF1, demonstrating a pronounced separation in community composition (Figure 5a). Further significance analysis revealed that *Bacillus* abundance was significantly higher in the BF3 group than in all other treatments, whereas *Priestia*, *Luteolibacter*, and *Rhizorhapis* showed significant enrichment, specifically in the BF1 group. *Microbulbifer* was identified as the core characteristic genus of the BF2 treatment, whereas *Sphingomonas* was relatively more abundant in the CK group (Figure 5b–g). The community difference in the BF3 treatment was characterized by a significant enrichment of *Bacillus* as its core feature, whereas the BF1 treatment demonstrated an enrichment effect on multiple functional genera, including *Priestia*, *Luteolibacter*, and *Rhizorhapis*.

## 4. Discussion

Continuous cropping obstacles have increasingly become a critical issue affecting crop yield and quality [62,63]. These challenges include the degradation of soil physical and chemical properties, alteration of soil microbial community structure, accumulation of autotoxins, weakened plant growth, and increased severity of diseases and pests [64]. These changes are primarily driven by the deterioration of the microbial habitat within the soil [7]. Therefore, modifying the soil microbial community structure represents a crucial approach for alleviating soil sickness syndrome in continuous cropping systems [65]. The introduction of beneficial microorganisms not only enhances plant growth and secondary metabolite accumulation but also fosters the development of healthier and more stable soil microbial communities, thereby advancing the sustainability and ecological viability of agricultural systems [66]. Despite substantial progress having been made in understanding the role of microbial inoculants in continuous cropping systems, comparative research remains limited on how functionally distinct single strains differentially affect soil properties, microbial community assembly, and crop performance, particularly in vegetable production systems such as eggplant. Most existing studies either focus on multi-strain consortia or evaluate microbial effects in isolation from integrated soil–plant–microbe feedbacks. Consequently, a clear understanding regarding how strain-specific functional traits distinctly shape rhizosphere community assembly, regulate soil nutrient cycling processes, and ultimately translate into varied crop physiological and quality outcomes under field conditions is still lacking. 

Microbial inoculants improved the soil environment in continuously cropped eggplant systems by increasing bacterial abundance, optimizing microbial community structure, and enhancing the presence of beneficial microorganisms, which is consistent with the core findings of Ahsan et al. [41], who reported that microbial consortia significantly altered the rhizosphere microbial composition of eggplant (*Solanum melongena L.*) and enriched beneficial PGPR taxa in continuous cropping soils. The rhizosphere microbial community plays a crucial role in supporting plant growth and sustaining health: *Leymus chinensis* enhances drought adaptability by recruiting beneficial rhizosphere microorganisms [67]; *Brassica oleracea* actively recruits beneficial microorganisms following pathogen infection, thereby enhancing its disease resistance [68]. The NMDS analysis revealed distinct separation of microbial communities among different inoculant treatments (Figure 2d), indicating that microbial inoculants significantly altered soil microbiome composition. This finding underscores the critical role of microbial amendments in shaping soil microbial community structure. Integrating and analyzing the taxon-specific changes in the rhizosphere soil microbial community under microbial inoculant application facilitates the identification and exploration of potential beneficial bacteria in the soil environment. The analysis of soil microbial community composition in this study revealed that at the phylum level, Pseudomonadota and Bacteroidota were the co-dominant phyla. This observation is consistent with several previous studies [69]. Pseudomonadota have been widely reported to participate in key soil biochemical processes, including nitrogen cycling and phosphorus transformation [24]. *Bacteroidota* species are frequently enriched in the plant microbiome and contribute to host fitness through multiple beneficial functions, including pathogen suppression [70]. At the genus level, the dominant taxa within Pseudomonadota were *Pseudomonas* and *Rhizobium*, which are well-known plant PGPR that enhance nutrient uptake and induce systemic resistance in eggplants [25,71]. One of the most pronounced changes observed in this study was the remarkable enrichment of *Bacillota* in the BF3 treatment, where its relative abundance was approximately 2.5 times that of the control (CK). Liu et al. [35] confirmed that synthetic Bacillus consortia could enhance plant biomass by 20–30% through bacterial social interactions, which aligns with our finding of BF3-mediated enrichment of native *Bacillus* and improved fruit quality. This phylum encompasses numerous beneficial microbes with stress-resistant traits, including the well-known genera *Bacillus* and *Priestia* [72]. This inoculated strain modifies the rhizosphere microenvironment by decomposing soil organic matter to release bioavailable nutrients and alleviating soil salinization stress, which creates a favorable niche for the growth of native Bacillota, taxa with well-characterized roles in soil nutrient cycling and plant health promotion. This process exemplifies how a single inoculated bacterial species can drive targeted changes in soil and rhizosphere microbial communities by reshaping key environmental filters, with these microbial shifts further translating into measurable improvements in soil fertility and eggplant fruit quality, a critical mechanistic link in continuous cropping systems ameliorated by microbial inoculants. Acidobacteriota exhibited the highest abundance in the BF1 treatment. This phylum is generally associated with soil organic matter accumulation and ecosystem stability [73], and its increase suggests that the BF1 treatment may contribute to the long-term health of the soil micro-ecosystem.

Soil organic matter, available nitrogen, and available nutrient contents are key indicators for assessing soil fertility and health. These nutrients not only contribute to the formation and stability of soil aggregates but also provide essential energy and material substrates for soil microbial activity, thereby enhancing the soil’s nutrient supply capacity. Furthermore, they can directly or indirectly improve plant nutrient uptake and utilization, ultimately supporting plant growth, development, and productivity. However, continuous cropping obstacles are often accompanied by soil nutrient imbalance and accumulation of salts and toxic compounds. In this study, only the BF3 treatment significantly increased the contents of SOC, SOM, AN, and TN (Figure 4), whereas BF1 and BF2 showed limited effects on soil physicochemical properties. This finding is consistent with the results of studies on tomato [74], where only one out of four microbial inoculant strains significantly improved soil carbon and nitrogen pools, highlighting the strong strain-specificity of microbial inoculants in regulating soil fertility. According to our RDA, the BF3 treatment formed a distinct cluster in the upper-left quadrant and showed strong positive correlations with key soil nutrients (SOC, TN, AN, TP), confirming its role in promoting nutrient transformation through selective enrichment of Bacillota. The seemingly contradictory pattern, in which the inoculant strain belongs to Actinomycetota while the enriched functional taxa are Bacillota, is likely attributable to the “keystone species effect” of *Zhihengliuella halotolerans*. This strain may secrete extracellular enzymes that decompose soil organic matter, releasing small-molecule nutrients that favor the growth of native Bacillota populations with high nutrient utilization efficiency [75]. However, this hypothesis requires further validation through pure culture co-cultivation experiments and metabolomic analysis of rhizosphere exudates. This phylum, which has diverse metabolic capabilities, directly participates in carbon and nitrogen cycling via extracellular enzyme production, organic matter mineralization, and nitrogen fixation [76]. The strain-specific effects observed in this study highlight that not all microbial inoculants can effectively improve soil fertility; instead, the selection of functional strains with strong nutrient cycling capabilities is critical for mitigating continuous cropping obstacles.

Microbial inoculants have been reported to enhance crop yield and quality in various crops. In tomato, the application of microbial inoculants significantly increased plant height and stem diameter, elevated sugar content by 25%, and improved overall fruit palatability [74,77]. According to Reis et al., inoculation of soybeans with *Bacillus amyloliquefaciens* and *Anabaena azollae* promotes both biomass and chlorophyll synthesis [78]. Organic fertilizer inoculated with *Bacillus velezensis* SQR9 increased pear yield [79]. In the present study, all three microbial inoculant treatments significantly improved eggplant yield and quality compared to the control. Plant height and stem diameter serve as direct indicators of plant growth and development. The results demonstrated that the BF1 treatment significantly increased both plant height and stem diameter, while the BF3 treatment led to significant increases in soluble sugar, soluble protein, and total amino acid content in eggplant fruits (Figure 1a–f). Microbial inoculants can regulate key processes in plant growth and development. The enhancement of vegetative growth was accompanied by higher crop yield, while the enrichment of beneficial microbes in BF3 treatment was associated with improved fruit quality indices (Figure 1).

Based on the findings of this study, microbial inoculants demonstrate clear potential for alleviating continuous cropping obstacles in facility-based eggplant cultivation. Specifically, BF1 and BF3 inoculants differentially regulated soil microbial community structure, leading to distinct outcomes: BF1 primarily promoted plant growth and yield, while BF3 improved soil fertility and fruit nutritional quality. However, this study has several limitations that should be acknowledged: (1) The conclusions are based on a single field experiment with a 4-month duration, and further validation across multiple growing seasons and soil types is required; (2) The specific functional mechanisms of the enriched microbial taxa were not elucidated in this study. Future research should integrate multi-omics techniques to elucidate functional gene expression and metabolic pathways, along with long-term field monitoring to validate ecological stability. These functionally defined microbial agents offer a novel strategy for soil health management in facility-based vegetable production. By reducing chemical inputs and improving the rhizosphere microenvironment, they are expected to support the transition of the facility eggplant industry toward sustainable intensification with reduced fertilizer and pesticide use, higher quality, and enhanced sustainability, ultimately contributing to the ecological management of soil degradation and the green development of agriculture.

## 5. Conclusions

This study confirmed that three distinct microbial inoculants exerted strain-specific effects on reshaping the eggplant rhizosphere microbiome. They enriched beneficial bacterial taxa, improved soil carbon and nitrogen cycling, and promoted plant growth, yield, and fruit quality to varying degrees. BF1 (*Bacillus zhangzhouensis*) significantly increased eggplant plant height by 32.1% and stem diameter by 28.7% compared with the control, boosting total yield by 142.4%. BF3 (*Zhihengliuella halotolerans*) selectively enhanced fruit quality, with total amino acid, soluble protein, and soluble sugar contents increased by 68.9%, 52.3%, and 41.2%, respectively; it also improved soil fertility, elevating SOC, TN, and AN by 13.73%, 18.03%, and 84.92% relative to CK. Meanwhile, BF3 enriched the relative abundance of beneficial *Bacillus* by 2.5-fold in the rhizosphere soil. BF2 (*Bacillus mobilis*) showed limited efficacy relative to the other two treatments. These findings highlight the value of targeted rhizosphere regulation via microbial inoculants, but further multi-crop, multi-soil, and long-term field studies are needed to develop precise strategies for sustainable agricultural intensification.

## Figures and Tables

**Table 1:** Alpha diversity indices of rhizosphere and non-rhizosphere microbial communities across different microbial inoculants.

Sample Type	Treatment	Dominance	Goods_Coverage	Observed_Features	Pielou_e	Simpson
Rhizosphere	CK	0.004 ± 0.001 ns	0.974 ± 0.002	2507 ± 122 b	0.869 ± 0.012 b	0.996 ± 0.001 ns
	BF1	0.003 ± 0.001 ns	0.969 ± 0.005	3010 ± 175 a	0.896 ± 0.016 a	0.997 ± 0.001 ns
	BF2	0.003 ± 0.000 ns	0.969 ± 0.003	2891 ± 62 a	0.885 ± 0.003 ab	0.997 ± 0.000 ns
	BF3	0.004 ± 0.002 ns	0.972 ± 0.001	2612 ± 80 b	0.866 ± 0.015 b	0.996 ± 0.002 ns
Non-rhizosphere	CK	0.003 ± 0.000 ns	0.976 ± 0.003	2586 ± 137 ns	0.889 ± 0.006 b	0.997 ± 0.000 ns
	BF1	0.003 ± 0.001 ns	0.973 ± 0.004	2814 ± 189 ns	0.897 ± 0.010 ab	0.997 ± 0.001 ns
	BF2	0.002 ± 0.000 ns	0.971 ± 0.003	2883 ± 147 ns	0.898 ± 0.005 a	0.998 ± 0.000 ns
	BF3	0.002 ± 0.000 ns	0.974 ± 0.004	2669 ± 116 ns	0.889 ± 0.003 b	0.998 ± 0.001 ns

Note: Intra-group significant differences were analyzed by Kruskal–Wallis nonparametric test followed by Mann–Whitney U test (α = 0.05). Different lowercase letters indicate significant differences within the same group (S/RS) at *p* < 0.05; “ns” means no significant difference within the group.

## Data Availability

The original contributions presented in this study are included in the article. Further inquiries can be directed to the corresponding author.
